# Genetic diversity of *Trypanosoma cruzi* strains isolated from chronic chagasic patients and non-human hosts in the state of São Paulo, Brazil

**DOI:** 10.1590/0074-02760220125

**Published:** 2022-11-11

**Authors:** Thiago Kury Moreno de Souza, Elizabeth Visone Nunes Westphalen, Sansão da Rocha Westphalen, Helena Hilomi Taniguchi, Carlos Roberto Elias, Gabriela Motoie, Ricardo Gava, Vera Lucia Pereira-Chioccola, Christina Terra Gallafrio Novaes, Noêmia Barbosa Carvalho, Edimar Alcides Bocchi, Fátima das Dores da Cruz, Mussya Cisotto Rocha, Samuel Katsuyuki Shinjo, Maria Aparecida Shikanai-Yasuda, Paola Andrea Ortiz, Marta Maria Geraldes Teixeira, José Eduardo Tolezano

**Affiliations:** 1 Instituto Adolfo Lutz, Centro de Parasitologia e Micologia, São Paulo, SP, Brasil; 2 Universidade de São Paulo, Faculdade de Medicina, Hospital das Clínicas, Divisão de Clínica de Moléstias Infecciosas e Parasitárias, São Paulo, SP, Brasil; 3 Universidade de São Paulo, Faculdade de Medicina, Hospital das Clínicas, Instituto do Coração, Unidade Clínica de Insuficiência Cardíaca, São Paulo, SP, Brasil; 4 Universidade de São Paulo, Faculdade de Medicina, Hospital das Clínicas, Laboratório de Imunologia (LIM 48), São Paulo, SP, Brasil; 5 Universidade de São Paulo, Faculdade de Medicina, Disciplina de Reumatologia, São Paulo, SP, Brasil; 6 Universidade de São Paulo, Faculdade de Medicina, Departamento de Moléstias Infecciosas e Parasitárias, São Paulo, SP, Brasil; 7 Universidade de São Paulo, Instituto de Ciências Biomédicas, Laboratório de Filogenia, Taxonomia e Diagnóstico de Tripanossomatídeos, São Paulo, SP, Brasil

**Keywords:** Chagas disease, host-pathogen interactions, molecular biology, Trypanosoma cruzi

## Abstract

**BACKGROUND:**

*Trypanosoma cruzi* shows an exuberant genetic diversity. Currently, seven phylogenetic lineages, called discrete typing units (DTUs), are recognised: TcI-TcVI and Tcbat. Despite advances in studies on *T. cruzi* and its populations, there is no consensus regarding its heterogeneity.

**OBJECTIVES:**

This study aimed to perform molecular characterisation of *T. cruzi* strains, isolated in the state of São Paulo, to identify the DTUs involved and evaluate their genetic diversity.

**METHODS:**

*T. cruzi* strains were isolated from biological samples of chronic chagasic patients, marsupials and triatomines through culture techniques and subjected to molecular characterisation using the fluorescent fragment length barcoding (FFLB) technique. Subsequently, the results were correlated with complementary information to enable better discrimination between the identified DTUs.

**FINDINGS:**

It was possible to identify TcI in two humans and two triatomines; TcII/VI in 19 humans, two marsupials and one triatomine; and TcIII in one human host, an individual that also presented a result for TcI, which indicated the possibility of a mixed infection. Regarding the strains characterised by the TcII/VI profile, the correlation with complementary information allowed to suggest that, in general, these parasite populations indeed correspond to the TcII genotype.

**MAIN CONCLUSIONS:**

The TcII/VI profile, associated with domestic cycles and patients with chronic Chagas disease, was the most prevalent among the identified DTUs. Furthermore, the correlation of the study results with complementary information made it possible to suggest that TcII is the predominant lineage of this work.

The heterogeneity of the protozoan parasite *Trypanosoma cruzi*, the etiologic agent of Chagas disease, concerning a variety of factors related to morphology, pathogenicity, virulence, genetic content, among others, has been observed for decades and has promoted numerous molecular epidemiology and population genetics studies. Consequently, this parasite has become one of the most notorious models regarding its evolution and population structure.^1^

In this scenario, a species identification method was developed in 2008, known as fluorescent fragment length barcoding (FFLB). This technique was initially tested for characterisation and differentiation of African trypanosomatids,^2^ and due to its efficiency, it was later used to determine phylogenetic lineages or discrete typing units (DTUs) of American trypanosomatids, having also shown robust results.^3^

The FFLB targets specific and polymorphic regions of the 18S and 28S ribosomal RNA (rRNA) genes, classified as 18S1, 18S3, 28S1 and 28S2. Through the polymerase chain reaction (PCR) assay, performed from a set of forward and reverse primers designed for each region, one of which is fluorolabelled, nucleotide sequences of variable lengths are amplified. Subsequently, the amplification products are submitted to capillary electrophoresis and observed, in an automatic sequencer, as fluorescent peaks - electropherograms - of variable size and intensity. Thus, the set of four peaks, one for each amplified region, theoretically constitutes a unique profile for each analysed species, known as a barcode.^2^

Currently, seven DTUs for *T. cruzi* are recognised: TcI-TcVI and Tcbat.^4^ ^-^ ^7^ Regarding the main DTU properties, TcI has a wide geographic distribution, extending from the southern United States to northern Argentina and Chile; is frequently isolated in sylvatic cycles, although it is also present in domestic cycles; is responsible for the transmission of Chagas disease in regions located north of the Amazon Basin^4^ ^,^ ^5^ ^,^ ^7^ ^-^ ^9^ and has considerable genetic diversity, with possible subdivisions within the lineage.^10^ ^-^ ^12^ ^)^ Regarding the other genotypes, TcII, TcV and TcVI are associated with domestic cycles and chronic chagasic patients in Southern Cone countries and Bolivia; TcIII and TcIV are found in rainforest sylvatic cycles;^4^ ^,^ ^5^ ^,^ ^7^ ^-^ ^9^ ^)^ and Tcbat was initially identified in bats^13^ ^,^ ^14^ and later found in humans.^15^ ^)^ Likewise, it is important to note that different DTUs can coexist on the same host.^16^ ^-^ ^18^

Despite the multiple advances in the study of *T. cruzi* and its populations, there is no consensus regarding its diversity.^6^ However, the occurrence of genetic recombination events between different lineages of this parasite may lead, at least partially, to the understanding of this process.^5^ ^,^ ^7^ ^,^ ^19^ ^-^ ^22^ In this sense, two major theories about the origin of DTUs, accepted by the scientific community, indicate, for example, that TcV and TcVI are hybrid lineages resulting from recombination between TcII and TcIII.^23^ ^,^ ^24^

The variety of triatomine vectors and mammalian hosts can play an important role in the distribution of DTUs, especially when related to environmental conditions. This correlation probably maintains the heterogeneity of *T. cruzi* and promotes the emergence of new variants through natural selection over time.^4^

Concerning the state of São Paulo (SP), it was already considered the territory with one of the highest prevalence of Chagas disease in Brazil, with the triatomine *Triatoma infestans* as the main transmitting species and with wide distribution in the region.^25^ ^,^ ^26^ After the implementation of successful actions to control vector transmission by local authorities between the 1950s and 1970s, the state’s epidemiological surveillance service has constantly monitored occurrences involving other triatomine species and the participation of sylvatic reservoirs in the process.^25^ In this scenario, *Panstrongylus megistus* stands out for the following factors: the remarkable ability to colonise artificial environments, with important reports of its finding in households and peridomiciles, especially in condominiums established in the urban areas of the metropolitan region of São Paulo; high infection rates by *T. cruzi*; and a great anthropophilia.^26^ ^,^ ^27^ This conjuncture of factors represents an alert for a possible recurrence of Chagas disease by vector transmission in the state, last reported more than 50 years ago.^28^

Contributing to epidemiological surveillance actions within the context of Chagas disease, the Instituto Adolfo Lutz (IAL), Laboratório Central de Saúde Pública do Estado de São Paulo (LACEN-SP), through the Centro de Parasitologia e Micologia (CPM), performs laboratory diagnosis of biological samples from different municipalities and health services and carries out field works in different regions of the state to study the ecoepidemiology of the disease by verifying the participation of sylvatic and domestic animals in its transmission. The CPM-IAL also meets demands for the identification of sylvatic or household triatomines and their possible natural infection by *T. cruzi*.

Considering the above, this study aimed to perform a molecular characterisation of *T. cruzi* strains isolated from biological samples analysed by the CPM-IAL from different host profiles, to identify their genotypes and expand knowledge about their diversity.

## SUBJECTS AND METHODS

*Samples* - Twenty-six samples positive for *T. cruzi* were selected from biological materials sent, processed and examined at CPM-IAL between 2014 and 2018. They originated from 21 chronic chagasic patients (Table I), mostly residing in SP, and periodically attended and followed by different health services - Serviço de Extensão ao Atendimento de Pacientes HIV/Aids (SEAP), Instituto do Coração (Incor) and Serviço de Reumatologia, Hospital das Clínicas, Faculdade de Medicina, Universidade de São Paulo (HCFMUSP) -, with different clinical conditions and positive laboratory tests for *T. cruzi*, by xenodiagnosis and blood culture, two sylvatic animals - marsupials of the *Didelphis albiventris* and *Philander opossum*species - from the São Paulo municipalities of Santa Fé do Sul and Ilhabela, respectively, with positive xenodiagnosis for *T. cruzi*, and three sylvatic triatomine specimens of the species *P. megistus*, collected from dwellings in the São Paulo municipalities of Taboão da Serra, Ilhabela and Itapecerica da Serra, with positive parasitological stool examinations for the presence of trypanosomatids and confirmatory real-time PCR test for *T. cruzi*.

**TABLE I:** Information from chronic chagasic patients whose blood samples were processed and examined at CPM-IAL

Patient	Age	Provenance (service)*	Provenance municipality (state)	Place of birth municipality (state)	Residence time in SP	Probable infection site municipality (state)	Landscape	Transmission route	Clinical condition	Treatment
1	55	HC-Incor	Guarulhos (SP)	Amargosa (BA)	No data	Amargosa (BA)	No data	Probably vector	Chronic phase - cardiac form	No data
2	70	HC-SEAP	São Paulo (SP)	Iguatu (CE)	51 years, arrived in 1971	Iguatu (CE)	Rural	Vector	Chronic phase - megaesophagus and cardiomyopathy	Yes, benznidazole in 2017
3	70	HC-Incor	Osasco (SP)	Santana dos Garrotes (PB)	No data	Santana dos Garrotes (PB)	No data	Probably vector	Chronic phase - cardiac form	No data
4	59	HC-Incor	São Paulo (SP)	Anápolis (GO)	No data	Anápolis (GO)	Rural	Probably vector	Chronic phase - cardiac form	No data
5	60	HC-SEAP	São Paulo (SP)	Cruz das Almas (BA)	47 years, arrived in 1975	Cruz das Almas (BA)	Rural	Vector	Chronic phase - megaesophagus	No
6	72	HC-Incor	São Paulo (SP)	João Ramalho (SP)	Stayed in SP	João Ramalho (SP)	No data	Probably vector	Chronic phase - cardiac form	No data
7	59	HC-Incor	São José dos Campos (SP)	São Benedito do Sul (PE)	No data	São Benedito do Sul (PE)	Rural	Probably vector	Chronic phase - cardiac form	No data
8	75	HC-SEAP	Araraquara (SP)	Rincão (SP)	Stayed in SP	Rincão (SP)	Rural	Vector	Chronic phase - megacolon, megaesophagus and cardiomyopathy	Yes, benznidazole in 2017, with parasitaemia negativation
9	54	HC-Incor	São Paulo (SP)	Castro Alves (BA)	No data	Castro Alves (BA)	Rural	Probably vector	Chronic phase - cardiac form	No data
10	61	HC-Incor	Salvador (BA)	Cruz das Almas (BA)	Non-resident	Cruz das Almas (BA)	Rural	Probably vector	Chronic phase - cardiac form	No data
11	48	HC-Reumatologia	São Paulo (SP)	Potosi (Bolivia)	First stay for 12 years, arrived in 1988; second stay since 2017	Potosi (Bolivia)	No details just a mud house with triatomines on site	Vector	Chronic phase - cardiac form; comorbidity - rheumatoid arthritis in immunosuppression, with intermittent parasitaemia	Yes, benznidazole for 57 days in 2016
12	63	HC-SEAP	São Paulo (SP)	Riacho de Santana (BA)	47 years, arrived in 1975	Riacho de Santana (BA)	Rural	Vector	Chronic phase - indeterminate form	No
13	69	HC-Incor	São Paulo (SP)	Ubaí (MG)	33 years, arrived in 1989	Ubaí (MG)	Rural	Probably vector	Chronic phase - cardiac form	No data
14	44	HC-Incor	São Paulo (SP)	Monte Azul (MG)	No data	Monte Azul (MG)	Rural	Probably vector	Chronic phase - cardiac form	No data
15	51	HC-SEAP	São Paulo (SP)	Sebastião Laranjeiras (BA)	32 years, arrived in 1990	Sebastião Laranjeiras (BA)	Rural	Vector	Chronic phase - indeterminate form	No
16	68	HC-SEAP	São Paulo (SP)	Conceição do Canindé (PI)	40 years, arrived in 1982	Conceição do Canindé (PI)	Rural	Vector	Chronic phase - megaesophagus and cardiomyopathy	No
17	59	HC-SEAP	São Paulo (SP)	Orobó (PE)	45 years, arrived in 1977	Orobó (PE)	Rural	Vector	Chronic phase - indeterminate form; reactivation with Chagas myelitis in 2008	Yes, benznidazole in 2008
18	70	HC-Incor	Guarulhos (SP)	Montes Claros (MG)	No data	Montes Claros (MG)	Rural	Probably vector	Chronic phase - cardiac form	No data
19	57, when died in 2021	HC-Reumatologia	São Paulo (SP)	Mundo Novo (BA)	33 years until his death in 2021, arrived in 1988	Mundo Novo (BA)	Rural	Vector	Chronic phase - cardiac form; comorbidity - polymyositis in immunosuppression, with intermittent parasitaemia; died in 2021	Incomplete - benznidazole for 12 days, in 2016, and discontinued due to adverse effect
20	62, when died in 2020	HC-Incor	São Paulo (SP)	Mundo Novo (BA)	No data	Mundo Novo (BA)	Rural	Probably vector	Chronic phase - cardiac form; died in 2020	No data
21	66	HC-SEAP	São Paulo (SP)	São Paulo (SP)	Stayed in SP	São Paulo (SP)	Urban	Transfusional	Haemophilic patient who received multiple transfusions over time; chronic phase - cardiac form	No

CPM-IAL: Centro de Parasitologia e Micologia-Instituto Adolfo Lutz. States: BA: Bahia; CE: Ceará; GO: Goiás; MG: Minas Gerais; PB: Paraíba; PE: Pernambuco; PI: Piauí; SP: São Paulo. * Serviço de Extensão ao Atendimento de Pacientes HIV/Aids (SEAP), Instituto do Coração (Incor) and Serviço de Reumatologia, Hospital das Clínicas, Faculdade de Medicina, Universidade de São Paulo (HCFMUSP).

*Strain isolation* - *T. cruzi* strains were isolated by blood cultures, xenocultures and cultures of digestive tracts of triatomines. Blood samples from patients were submitted to blood cultures; xenocultures were processed from triatomines of the species *Rhodnius neglectus*, bred in the CPM-IAL and used in field works involving xenodiagnosis of the marsupials, and the digestive tracts of *P. megistus* specimens were grown in axenic cultures.

*Blood culture* - The blood culture technique was used^29^ with modifications. Amounts of 10 to 30 mL of venous blood were collected from each patient in heparin tubes. The blood samples were transferred to 50 mL tubes and centrifuged at 2,410 x *g* at 4ºC for 10 minutes. The sediment of red blood cells, containing the buffy coat, was washed once with 10 mL liver infusion tryptose (LIT) medium, centrifuged at 2,410 x *g* at 4ºC for 20 minutes and resuspended in 10 mL of fresh medium. Then, this suspension was distributed to six 15 mL tubes containing 4 mL LIT each - about 2 mL suspension/tube - and incubated in a biochemical oxygen demand (BOD) system at 25ºC. Amounts of 10 µL suspension from each tube were examined monthly for 120 days in a common optical microscope at 400X magnification.

*Xenoculture/culture of the digestive tract of triatomines* - The following technique was used^30^ with modifications. The digestive tracts of triatomines, positive for *T. cruzi*, were cultured in tubes containing a biphasic medium composed of a slanted solid phase of 6 mL blood agar base (BAB) or Ducrey agar from rabbit blood and a liquid phase of 2 mL brain heart infusion (BHI) broth treated with 200 µg/mL gentamicin. The tubes were incubated in a BOD system at 25°C, with the first assessment of the material performed at 4th day of culture, in a common optical microscope at 400X magnification. Subsequent assessments were performed once a week, for 30 days.

*Maintenance and multiplication of the isolated strains* - Once positivity for *T. cruzi* was confirmed, the cultures remained in a BOD system at 25°C and the parasites were subjected to periodic repetitions in LIT medium for maintenance and multiplication. Additionally, aliquots of 1 mL of each culture were cryopreserved at -196°C. The *T. cruzi* strains were kept in these systems until the moment of use in the methodologies for molecular characterisation performed in this work.

*DNA extraction from T. cruzi-positive cultures* - Procedure performed with the QIAamp^®^ DNA Mini Kit (Qiagen, Valencia, USA) commercial kit. Aliquots of 1 mL, obtained from each *T. cruzi*-positive culture, were transferred to 1.5 mL tubes and centrifuged at 6,000 x *g* for 1 minute. The supernatants were removed and the pellets were resuspended in 1 mL phosphate buffered saline (PBS) and centrifuged at 6,000 x *g* for 1 minute. After this step, 200 µL AL buffer and 20 µL proteinase K were added to each tube, followed by vortexing and incubation in a water bath at 56°C for 30 minutes. Subsequently, DNA purification was performed according to manufacturer’s recommendations.

*Quantification and storage of extracted DNAs* - The concentrations and quality of the extracted DNAs were evaluated by spectrophotometry in Nanodrop™ ND-100 (Thermo Scientific, Waltham, USA) at 260 and 280 nm. The DNAs were stored at -20ºC until use.

*Molecular characterisation by FFLB* - The FFLB methodology was used^2^ with modifications. PCR assays were performed separately for each of the four primer pairs, with one fluorolabelled primer for each region to be amplified,^2^ to a final volume of 15 µL containing 0.5 µL (100 pmoles/µL) forward and reverse primers, 13 µL Invitrogen™ Platinum™ PCR SuperMix (Life Technologies, Carlsbad, USA) and 1 µL DNA template, extracted from each *T. cruzi*-positive culture. Each PCR assay ran with one positive control for trypanosomatids - Tcon025E - and one negative, without DNA, to ensure the reliability of the results. The reactions were carried out in a Mastercycler™ Nexus Gradient (Eppendorf, Hamburg, Germany) thermocycler, according to the following conditions: 35 cycles of 45 seconds at 95°C, 30 seconds at 62°C and 60 seconds at 72°C, with initial denaturation and final extension of 95°C for 3 minutes and 72°C for 10 minutes, respectively. PCR products, initially, were submitted to electrophoresis in 2% (w/v) agarose gel in Tris-acetate-ethylenediamine tetraacetic acid (TAE) buffer, stained with GelRed^®^ (Biotium, Fremont, USA) and visualised under ultraviolet (UV) transillumination. After having confirmed the presence of bands indicating positivity for trypanosomatids, PCR products were prepared in 96-well plates by adding 9 µL Applied Biosystems™ GeneScan™ 500 ROX™ Size Standard (Life Technologies, Warrington, UK) molecular weight marker with formamide, 1: 30 dilution, and 1.5 µL of amplified product/well followed by vortexing. The plate analysis was processed in an Applied Biosystems™ 3500 Genetic Analyzer (Life Technologies, São Paulo, Brazil) automatic sequencer and the peaks produced, referring to the amplified fragments, were analysed by the Applied Biosystems™ GeneMapper™ (Life Technologies, Foster City, USA) software. Subsequently, the barcodes obtained were compared to those from the original study^3^ and from later research, carried out at the Laboratório de Filogenia, Taxonomia e Diagnóstico de Tripanossomatídeos of the Instituto de Ciências Biomédicas da Universidade de São Paulo (ICB-USP), also responsible for the better standardisation of the FFLB,^31^ ^,^ ^32^ to identify the DTUs of the *T. cruzi* strains studied.

*Correlation of results with complementary information -* The results of molecular characterisation were complemented with information from patients’ charts, presented in Table I, from review works that include distribution maps and graphs of *T. cruzi* DTUs,^4^ ^-^ ^7^ ^,^ ^9^ ^,^ ^33^ and a previous research study^32^ to enable better discrimination between the identified genotypes and provide greater robustness to the study’s findings.

*Plotting the results in a distribution map* - The identified DTUs were plotted on a distribution map by the My Maps (Google LLC) app, according to information from each host.

*Ethics -* This study was developed under a research project involving human beings, approved by the Ethics Commission for Analysis of Research Projects (CAPPesq) of HCFMUSP on April 20, 2016 (protocol nº 1043/07), which is in agreement with the Helsinki Declaration of 1975, as revised in 1983.

## RESULTS

*Molecular characterisation by FFLB* - The base pairs (bp) size of the amplified fragments and the per-host *T. cruzi* genotypes identified are shown in Table II. It was possible to identify TcI in four hosts (two humans and two triatomines), TcII/VI in 22 hosts (19 humans, two marsupials and one triatomine) and TcIII in one human host. In the latter case, the individual also presented a result for TcI, which indicated the possibility of a mixed infection. Figure shows the distribution map of *T. cruzi* DTUs identified in this study, according to the probable infection sites for human hosts and the provenance of the infected hosts belonging to sylvatic fauna. It is important to note here that the DTU identified in patient 21, TcII/VI, may have originated in another municipality or, more broadly, in another state, given that this individual is haemophilic and was infected via blood transfusion. Moreover, the impossibility of determining the origin of the infected blood donor did not allow the definition of this specific case; hence, the identified DTU was plotted only to indicate the probable infection site of the mentioned patient.

**TABLE II:** Fragments amplified for 18S1, 18S3, 28S1 and 28S2 regions and the identified DTUs per host

Host	Fragment length (bp)				Results
	18S1	18S3	28S1	28S2	
Patient 1	299, 300, 301	241, 242	349, 350	212, 213	TcII/VI
Patient 2	300	241, 242	347, 348, 349, 350	212, 213	TcII/VI
Patient 3	300	241, 242	348, 349, 350, 351	212, 213	TcII/VI
Patient 4	299, 300	241, 242	349, 350	212, 213	TcII/VI
Patient 5	300	241, 242	346, 347	212, 213	TcII/VI
Patient 6	299, 300	241, 242	346, 347, 349, 350, 351	212, 213	TcII/VI
Patient 7	300	241, 242	348, 349, 350	212, 213	TcII/VI
Patient 8	299, 300	241, 242	●●●	212, 213	TcII/VI
Patient 9	299, 300	241, 242	346, 347, 348, 350	212, 213	TcII/VI
Patient 10	299, 300	241, 242	347, 348, 349, 350	212, 213	TcII/VI
Patient 11	304, 305	245, 246	338, 339, 340, 341	196, 197	TcI
Patient 12	299, 300	241, 242	347, 348, 349, 350	212, 213	TcII/VI
Patient 13	300	241, 242	348, 349, 350	212, 213	TcII/VI
Patient 14	299, 300	241, 242	349, 350	212, 213	TcII/VI
Patient 15	300	241, 242	347, 348, 349, 350	211, 213	TcII/VI
Patient 16	300	241, 242	347, 348, 351, 352	212, 213	TcII/VI
Patient 17	299, 305	236, 244, 245	333, 334, 336, 338, 339	189, 196, 197	TcI and TcIII
Patient 18	299, 300	241, 242	347, 348, 349, 350	212, 213	TcII/VI
Patient 19	300	241, 242	346, 348, 349, 350	212, 213	TcII/VI
Patient 20	300	241, 242	346, 347, 349, 350	212, 213	TcII/VI
Patient 21	299, 300	241, 242	347, 348, 349, 350	212, 213	TcII/VI
*Philander opossum* - Ilhabela	300	241, 242	348, 349	212, 213	TcII/VI
*Didelphis albiventris* - Santa Fé do Sul	299, 300	241, 242	347, 348, 349, 350	212, 213	TcII/VI
*Panstrongylus megistus* - Taboão da Serra	299, 304, 305	244, 245	333, 335, 336, 338, 339, 340	196	TcI
*Panstrongylus megistus* - Ilhabela	299, 300	241, 242	348, 349, 350, 351	212, 213	TcII/VI
*Panstrongylus megistus* - Itapecerica da Serra	299, 305	244, 245	332, 334, 335, 338, 339, 340	196, 197	TcI

bp: base pairs; ■: fragments corresponding to TcI; ■: fragments corresponding to TcII/VI; ■: fragments corresponding to TcIII; ■: fragments that are common to different discrete typing units (DTUs); ●●●: no fragments were detected.

**Figure:**
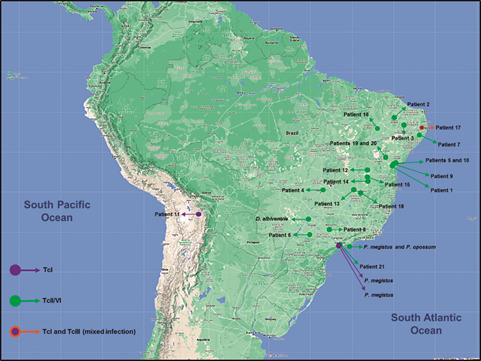
The distribution map of *Trypanosoma cruzi* discrete typing units (DTUs) identified in this study according to the probable infection sites for human hosts (patients) and the provenance of the infected sylvatic hosts (marsupials and triatomines). Bar = 100 km.

*Correlation of results with complementary information* - The correlation of molecular characterisation results with information from patients’ charts, reviews containing distribution maps and graphs of *T. cruzi* DTUs^4^ ^-^ ^7^ ^,^ ^9^ ^,^ ^33^ and a previous research study^32^ ^)^ made it possible to better discriminate TcII from TcVI and allowed to suggest that, predominantly, the strains identified by the TcII/VI profile indeed correspond to TcII.

## DISCUSSION

*T. cruzi* contemplates an exuberant genetic diversity^4^ ^-^ ^9^ and a complex life cycle, with the latter involving extracellular proliferation and differentiation in hematophagous insect - triatomine vectors - and intracellular proliferation and differentiation in a range of mammalian species. The constant transition from an invertebrate host to a vertebrate one sets different pressures, either by the immune response of these hosts or by the new environment of parasite development.^34^ ^,^ ^35^ Despite this factor being considered in the attempt to understand the biological characteristics intrinsic to the parasite, there is no consensus, so far, that explains the heterogeneity observed in its populations.^6^ However, the emergence of population variants can be derived by the occurrence of genetic recombination events between different *T. cruzi* lineages throughout the evolutionary process.^5^ ^,^ ^7^ ^,^ ^19^ ^-^ ^22^ In this context, there are two major theories, recognised by the scientific community, that propose that the TcV and TcVI genotypes originate from recombinations between the TcII and TcIII genotypes.^23^ ^,^ ^24^ ^)^ It is also accepted that TcI and TcII are pure lineages that evolved separately from a common ancestor millions of years ago.^4^ ^,^ ^9^ ^,^ ^24^ ^,^ ^36^

The theories reported above can largely explain the difficulty in differentiating parental lines from hybrid lines when using molecular techniques to identify DTUs of *T. cruzi* strains. This difficulty was corroborated in the results of this work, in which TcII and TcVI could not be discriminated by FFLB, despite the use of highly sensitive primers, as they share DNA fragments of the same size. This limitation and others, exposed by the characterisation of *T. cruzi* strains by FFLB, were previously addressed by different works.^3^ ^,^ ^31^ ^,^ ^32^ However, this molecular technique has advantages compared to other PCR-based trypanosomatids identification methods. Among them are the speed and sensitivity of the method, as it can amplify relatively small regions of DNA, and the capabilities to detect fluorescence and differentiate mixed infections. Furthermore, this method can discriminate species and a range of lineages from the same set of primers and facilitate epidemiological studies and large-scale investigations.^2^ ^,^ ^3^ ^,^ ^32^

In the group of strains studied, this work verified the predominance of the TcII/VI profile, represented by 22 hosts, mostly humans. Among the 19 patients within this profile, two were affected by the indeterminate form, 13 by the cardiac form, one by the digestive form - megaesophagus - and three by the mixed form, the latter concomitantly involving cardiomyopathy and mega syndrome. These data are in accordance with the information described in the literature, which relate the transmission pattern and characteristic host profile for these DTUs, to domestic transmission cycles and patients with chronic Chagas disease, respectively, in addition to the variety of clinical pictures promoted, encompassing the cardiac, digestive and mixed forms.^5^ ^,^ ^7^ ^,^ ^9^ ^)^ Complementing the results of molecular identification of *T. cruzi* strains isolated from humans, patients 11 and 17, probably infected in Bolivia and the Brazilian Northeast Region, respectively, harboured TcI, the first being affected by the cardiac form and the second by the indeterminate form. In patient 17, TcIII was also detected, suggesting a mixed infection.

As for the strains isolated from sylvatic fauna, here comprised between marsupials and triatomines, the results corroborate the classical epidemiology of Chagas disease. Specifically, the sylvatic transmission cycles in an environment of balance between vectors and hosts, followed by disturbances and alterations of these cycles by the introduction of buildings and homes in forested areas, and the consequent modification of the transmission dynamics to a domestic or peridomestic pattern.^37^ ^,^ ^38^ In this scenario and concerning the TcI genotype, the two specimens of *P. megistus* from the municipalities of Taboão da Serra and Itapecerica da Serra, which harboured parasites characterised by this DTU, were found and collected inside households located in areas of environmental preservation. A prominent feature of the condominiums that comprise these households is the spatialisation of the residences, which are more distant from each other when compared to urban areas. This environmental transformation can favour the manifestation of synanthropic behaviours by sylvatic reservoirs and the encounter of triatomines inside and around the dwellings.^27^

The TcII/VI profile, identified in the strains isolated from marsupials *D. albiventris* and *P. opossum* from the municipalities of Santa Fé do Sul and Ilhabela, respectively, and from one of the *P. megistus* specimens, also from Ilhabela, can be understood by two factors: a) the environment modification, mentioned above, changed the dynamics of transmission cycles and promoted the inclusion of human hosts in this process; and b) synanthropy, characteristic of these sylvatic animals, favoured the displacement of the vector insect to the new location in which these mammals settled, for being their food source. Thus, both animals and triatomine vectors were able to access the urban environment, modifying the cycle and favouring a new transmission pattern.^37^ ^,^ ^38^

Such epidemiological aspects raise concerns since a colonisation of triatomines, especially the *P. megistus* species, has been observed in SP, placing the population at risk of Chagas disease by natural transmission,^27^ a phenomenon last reported more than 50 years ago in the region.^28^ This scenario is mainly verified in households and peridomiciles in municipalities following the Trecho Oeste and Trecho Sul of the Rodoanel Metropolitano Mário Covas, in locations with natural forest reserves. The municipalities comprised are: Carapicuíba, Cotia, Embu das Artes, Itapecerica da Serra, Osasco, Ribeirão Pires, Santana do Parnaíba, Santo André, São Bernardo do Campo, São Paulo e Taboão da Serra.^39^ ^)^ Furthermore, it is important to highlight that the presence of didelphid marsupials in areas of the metropolitan region, close to dwellings, is high, given their ability to adapt to the urban environment. These sylvatic animals have high rates of natural infection by *T. cruzi*, and probably contribute to maintaining the circulation of the parasite in the region.^27^

The complementation of the molecular results with information obtained from patients’ charts, reviews that include distribution maps and graphs of *T. cruzi* DTUs^4^ ^-^ ^7^ ^,^ ^9^ ^,^ ^33^ and a previous research study,^32^ made it possible to establish certain discrimination between the TcII and TcVI genotypes, allowing to suggest that, in general, the strains characterised by the TcII/VI profile indeed correspond to TcII. This statement can be further justified if we consider that most of the hosts in this study are humans who, although reside in SP - except for patient 10 - are predominantly from cities in the Brazilian Northeast Region, where there are no reports so far of the TcVI occurrence,^32^ and that such individuals were probably infected there. In addition, according to elements provided by the reviews consulted,^4^ ^-^ ^7^ ^,^ ^9^ ^,^ ^33^ it is rare or unlikely to find TcVI within the regions that comprise the municipalities of the other hosts approached here, in contrast to the high frequency of TcII. However, future works should promote the differentiation of hybrid DTUs from their evolutionary predecessors to obtain more precise and definitive results through other molecular techniques.

It is important to point out that, except for the technical limitations for discriminating between TcII and TcVI, the FFLB identified the genotypes of all strains isolated in culture media. However, artificial conditions in culture media or even *in vivo* experimental models represent potential selective pressures for the predominance or elimination of a given parasite population.^6^ ^,^ ^7^ This feature had already been observed from the analysis of electrophoretic profiles of DNA from different *T. cruzi* strains. In one of these experiments, *T. cruzi* strains were isolated from human hosts by blood culture and inoculated into murine models. After inoculation, these animals were followed for a period of 2 years by schizodeme analysis and the results showed that there were cases in which the initially observed electrophoretic profiles were replaced by others over time, indicating selectivity by the animals’ organism.^40^

In other words, it cannot be said that the samples used here and submitted to culture originally harboured a single *T. cruzi* population. It would be necessary to extract the DNA from the original biological material - blood from vertebrate hosts and triatomine faeces - and proceed with molecular identification to verify the coexistence between different DTUs.

Given the results, we could conclude that: the molecular characterisation of *T. cruzi* strains identified the TcI, TcII/VI and TcIII genotypes; the TcII/VI profile, associated with domestic cycles and patients with chronic Chagas’ disease, was the most prevalent among the identified genotypes and whose derivation was mostly from human hosts; the complementation of molecular results with additional information allowed us to suggest that TcII is the predominant lineage of this research; two human hosts harboured TcI (patients 11 and 17), where there was a suggestive result for mixed infection in one of them, involving TcI and TcIII (patient 17); and of the strains isolated from sylvatic fauna, two were characterised as TcI (*P. megistus* specimens from Taboão da Serra and Itapecerica da Serra) and three as TcII/VI (*P. megistus* and *P. opossum* from Ilhabela and *D. albiventris* from Santa Fé do Sul) associated with sylvatic and domestic cycles, respectively.

This work having identified the DTUs of *T. cruzi* strains isolated from different host profiles, contributes to the epidemiology of Chagas disease in SP, reinforces the attention to the possible recurrence of this disease in the region, from natural transmission by triatomine vectors, and provides a basis for studies on the genetic diversity of this parasite.
